# Gaucher's disease with myocardial involvement in pregnancy

**DOI:** 10.1590/S1516-31802002000300008

**Published:** 2002-05-02

**Authors:** Maria Regina Torloni, Kátia Franco, Nelson Sass

**Keywords:** Gaucher's disease, Pregnancy, Myocardial involvement, Heart failure, Doença de Gaucher, Gravidez, Miocardiopatia, Insuficiência cardíaca

## Abstract

**CONTEXT:**

Described originally in 1882, Gaucher's disease is the most prevalent of storage disorders. This autosomal recessive disease is caused by a defective gene responsible for coding the beta-glucosidase enzyme, essential in the hydrolysis of glucosylceramide in glucose and ceramide. The accumulation of glucosylceramide in the lysosomes of the reticuloendothelial system produces a heterogeneous clinical picture with neurological involvement, liver and spleen enlargement, hematological disorders and bone lesions.

**CASE REPORT:**

Two pregnancies of a patient with Gaucher's disease are presented. The patient, who had been asymptomatic following earlier splenectomy, developed congestive heart failure due to myocardial involvement at the beginning of her first pregnancy, and responded to conservative treatment. In spite of this complication and also chronic anemia, hepatomegaly and ascites due to portal hypertension, the patient had two successful pregnancies with good perinatal results. No hemorrhagic complications were observed.

## RESUMO

**CONTEXTO:**

Descrita originalmente em 1882, a doença de Gaucher é a mais prevalente das doenças de depósito. Esta doença autos-sômica recessiva é causada por um defeito no gene que codifica a enzima beta-glucocerebrosidase, essencial na hidrólise de glucosilceramida em glicose e ceramida. O acúmulo da glucosilceramida dentro dos lisossomos das células do sistema retículoendotelial produz um quadro clínico variável caracterizado por déficit neurológico, hepatosplenomegalia, anomalias hematológicas e lesões ósseas.

**RELATO DO CASO:**

As duas gestações de uma paciente com doença de Gaucher são descritas. A gestante, assintomática desde sua esplenectomia, apresentou insuficiência cardíaca por miocardiopatia no início de sua primeira gravidez, respondendo ao tratamento conservador. Apesar desta complicação, da anemia crônica, de hepatomegalia e ascite decorrente de hipertensão portal, a paciente evoluiu bem em duas gestações e o resultado perinatal foi bom. Não observamos complicações hemorrágicas.

## INTRODUCTION

Described originally by Philippe Gaucher in 1882, Gaucher's disease is the most prevalent of storage disorders. This autosomal recessive disease is caused by a defective gene located in the short arm of chromosome 1, responsible for coding the beta-glucosidase enzyme, essential in the hydrolysis of glucosylceramide in glucose and ceramide. The accumulation of glucosylceramide in the lysosomes of the reticuloendothelial system produces a heterogeneous clinical picture with neurological involvement, liver and spleen enlargement, hematological disorders and bone lesions. It affects 1:40,000 to 1:60,000 individuals in the US and occurs 30 times more frequently among the Ashkenazi Jews. Its real incidence in Brazil is unknown. It is classified into three types according to the degree of neurological involvement, but only patients with type I reach reproductive age. The diagnosis is based on biochemical assays of enzyme activity or on liver, spleen or bone marrow biopsies, which reveal typical "Gaucher's cells" (large, pale histiocytes with a peripheral nucleus). There is still some controversy about the reciprocal effects of Gaucher's disease and pregnancy. We present the course and outcome of two pregnancies in a patient with Gaucher's disease who developed heart failure due to the disease.

## CASE REPORT

The patient, a 23-year-old black Catholic, presented at the hospital in the 14^th^ week of her first pregnancy. She had had a history of anemia, liver and spleen enlargement and urinary tract infections since early childhood, having been diagnosed with Gaucher's disease following a liver biopsy when she was 12. She underwent splenectomy at the age of 17 and thereafter abandoned follow-ups, since she was apparently doing well.

**Figure 1:**
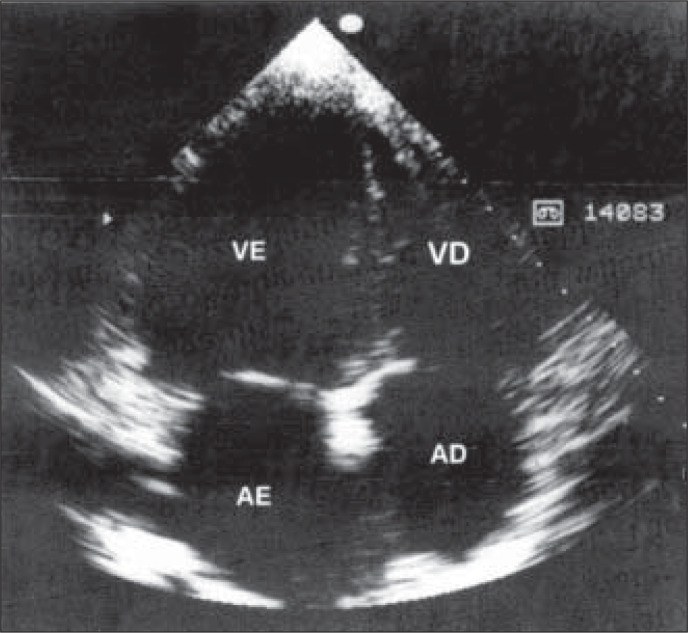
Two-dimensional echocardiogram in the 4-chamber view, showing general dilatation.

**Figure 2:**
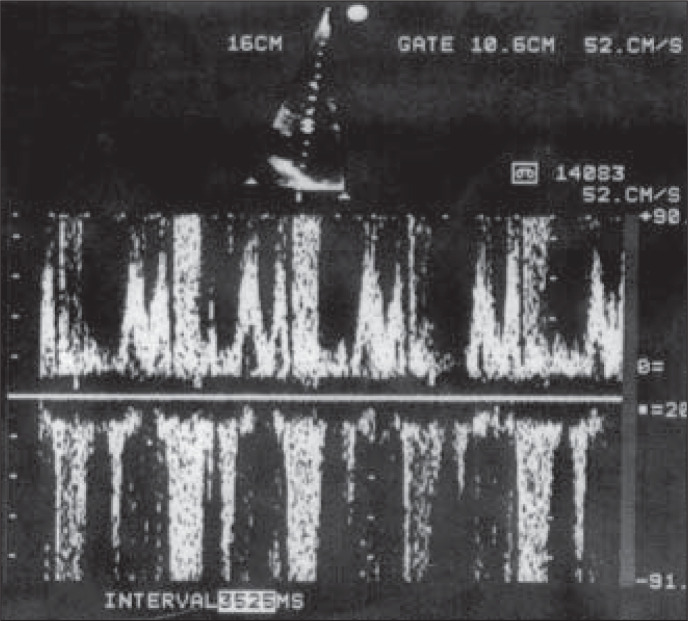
Echocardiogram showing mitral regurgitation.

**Figure 3:**
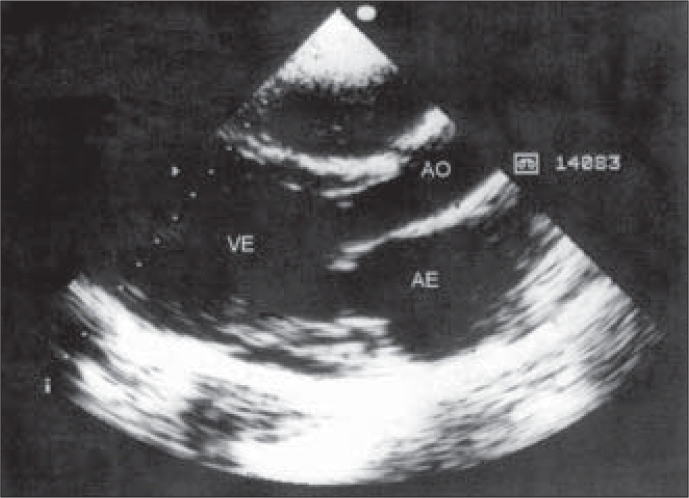
Echocardiogram showing outlet of left ventricle with dilated chambers.

On admission she was dyspneic, pale (hemoglobin of 9.5 g/dl), with a hard non-tender liver edge palpable 20 cm below the costal margin, jugular distention and lower limb edema. On the chest X-ray there was a global cardiomegaly and pulmonary congestion. Echocardiography revealed a dilated myocardium with moderate mitral and tricuspid regurgitation (Figures 1, 2 and 3). She responded well to digoxin, diuretics and iron supplements, being discharged to prenatal care. She was again admitted in her 28^th^ week for fetal evaluation due to the aged aspect of the placenta on ultrasound (grade III). She had weekly cardiotocography, Doppler and ultrasound examinations (all normal) and maintained chronic anemia (9 g/dl) with normal platelet counts. She had an episode of urinary infection that required a prolonged course of antibiotics. Spontaneous labor occurred at 40 weeks, and she underwent cesarean section due to fetal distress. Bleeding was normal and the postpartum period was uneventful, with the patient being discharged on the 5^th^ day, still on digoxin and diuretics. The newborn weighed 2580g, had normal Apgar scores (8 and 9) and was discharged with the mother.

The patient presented again when she was 30, in the 20^th^ week of her second pregnancy, asymptomatic albeit using digoxin since the first pregnancy. She was pale (hemoglobin 7.5 g/dl) and her liver was enlarged (15 cm below the costal margin) and non-tender. She was followed up as an outpatient throughout pregnancy and maintained platelet counts of between 120,000 and 180,000/mm^3^, with chronic anemia that did not require transfusion. Maternal ascites was detected on the 39^th^ week during ultrasound and the fetus presented hydramnios and increased resistance of the umbilical artery. She underwent cesarean section and tubal ligation with no complications during or after surgery. The newborn weighed 3050g, had Apgar scores of 9 and 10 and did well until discharge with his mother on the 3^rd^ postpartum day. Pathology reports on the placentas revealed single umbilical arteries in both pregnancies and no Gaucher cells.

## DISCUSSION

Until 1960 the few case reports of Gaucher's disease in pregnancy suggested that these women tended to be sterile, had more miscarriages and neonatal deaths and were exposed to a greater risk of maternal death. Based on these papers and on the fear of transmission of the disease to their children, these patients were advised not get pregnant and offered therapeutic abortions and sterilization.^1^ In the following thirty years a series of publications presented good maternal and perinatal results, indicating that pregnancy did not exacerbate Gaucher's disease.^2^

The vast majority of pregnancies go well but occasionally, severe hemorrhagic complications may occur during labor and delivery due to thrombocytopenia. These complications are rare in patients undergoing splenectomy before pregnancy, but it is recommended that all women with Gaucher's disease be carefully monitored (hemogram and coagulogram) during pregnancy. Chronic moderate normocytic anemia is frequent during pregnancy and is usually attributed to splenic sequestration of red blood cells or Gaucher's cells involvement in bone marrow. Some^3^ recommend treatment of anemia and thrombocytopenia with steroids due to their myelogenic effect.

Hepatomegaly is a common finding during pregnancy and is presumably due to hepatic infiltration by Gaucher's cells and ensuing fibrosis and compression of sinusoid vessels, which can also lead to portal hypertension and ascites, a sign that we observed in this patient's 2^nd^ pregnancy.

Myocardial infiltration by Gaucher's cells can result in contractile impairment, particularly of the left ventricle. On reviewing national and international literature we found a few cases of heart involvement in Gaucher's disease in adults^4^ and were unable to find any cases related to pregnancy. Our patient responded to conventional treatment with diuretics and digitalis, but remained chronically dependent on the medication. A new treatment option has emerged recently with enzyme replacement therapy using alglucerase and imiglucerase, which are drugs obtained from placental extraction and recombinant DNA technology. The effects on adults and children are promising, with regression of spleen and liver enlargement. Good results were observed in a study on a few patients (6 cases) treated with enzyme replacement therapy during pregnancy.^5^ Unfortunately, the high cost of these drugs limit their use in developing countries.

Using conservative symptomatic treatment we had good maternal and perinatal results in the two pregnancies of our patient with Gaucher's disease. Our results are in agreement with and confirm similar findings published in the literature over the last few decades. We have reported the first case of Gaucher's disease with myocardial involvement in pregnancy. The heart failure responded well to conventional (digoxin and diuretics) treatment.
